# Detailed Structural Characterization of the Lipooligosaccharide from the Extracellular Membrane Vesicles of *Shewanella vesiculosa* HM13

**DOI:** 10.3390/md18050231

**Published:** 2020-04-27

**Authors:** Rossella Di Guida, Angela Casillo, Fumiaki Yokoyama, Jun Kawamoto, Tatsuo Kurihara, Maria Michela Corsaro

**Affiliations:** 1 Department of Chemical Sciences, University of Naples “Federico II”, Complesso Universitario Monte S. Angelo, Via Cintia 4, 80126 Naples, Italy; ross.diguida@gmail.com; 2 Task Force Blue Italian Growth BigFedII, University of Naples “Federico II”, 80126 Naples, Italy; 3 Institute for Chemical Research, Kyoto University, Uji, Kyoto 611-0011, Japan; yokoyama@mbc.kuicr.kyoto-u.ac.jp (F.Y.); jun_k@mbc.kuicr.kyoto-u.ac.jp (J.K.); kurihara@scl.kyoto-u.ac.jp (T.K.)

**Keywords:** *Shewanella vesiculosa*, cold-adapted bacterium, extracellular membrane vesicles, mass spectrometry, lipid A, core oligosaccharide

## Abstract

Bacterial extracellular membrane vesicles (EMVs) are membrane-bound particles released during cell growth by a variety of microorganisms, among which are cold-adapted bacteria. *Shewanella vesiculosa* HM13, a cold-adapted Gram-negative bacterium isolated from the intestine of a horse mackerel, is able to produce a large amount of EMVs. *S. vesiculosa* HM13 has been found to include a cargo protein, P49, in the EMVs, but the entire mechanism in which P49 is preferentially included in the vesicles has still not been completely deciphered. Given these premises, and since the structural study of the components of the EMVs is crucial for deciphering the P49 transport mechanism, in this study the complete characterization of the lipooligosaccharide (LOS) isolated from the cells and from the EMVs of *S. vesiculosa* HM13 grown at 18 °C is reported. Both lipid A and core oligosaccharide have been characterized by chemical and spectroscopic methods.

## 1. Introduction

Extracellular membrane vesicles (EMVs) are spherical membrane-bound particles produced during cell growth by a wide variety of Gram-negative bacteria [1]. In the biogenesis of these EMVs, a small portion of the bacterial outer membrane (OM) bulges away from the cell. For this reason, in Gram-negative bacteria, they are primarily composed of lipopolysaccharides (LPSs), membrane phospholipids, and outer membrane proteins [2]. LPSs and membrane phospholipids are the major components of EMVs for their high surface–volume ratio. In addition, EMVs can contain different cargoes such as DNA, RNA, many different periplasmic and cytoplasmic proteins, and toxins. These molecules and cargoes confer to EMVs a role in diverse physiological and pathological functions [3]. These include transfer of proteins between bacterial cells, cell-to-cell signaling, biofilm formation, and bacterial survival [4,5]. Given their intrinsic characteristics, EMVs have many potential applications including drug delivery vehicles, scaffolding of enzymatic reaction (making EMVs natural nanoreactors), vaccines, and adjuvants [1,6,7]. It has been shown that growth temperature influences the quantity and morphology of EMVs produced by *Shewanellalivingstonensis* NF22T, a psychrotolerant Antarctic bacterium [8,9]. Moreover, a proteomic analysis revealed that the proteins in the EMVs of this bacterium are mainly involved in nutrient sensing and bacterial survival.

Very recently, it was reported that the cold-adapted Gram-negative bacterium *Shewanella vesiculosa* HM13 (formerly *Shewanella* sp. HM13) is able to produce a large amount of EMVs carrying a single major cargo protein—P49 [10]. The function of P49 is currently unknown and not predictable based on its sequence. However, P49 is expected to be useful as a carrier to deliver a foreign protein to EMVs in the recombinant protein production system by using this strain as the host. In the transportation of P49 to EMVs, it is reasonable to assume that components of the outer membrane, such as LPSs, play a role because disruption of the gene putatively involved in the synthesis of surface glycolipids caused dissociation of P49 from EMVs [11]. LPSs are amphiphilic macromolecules generally composed of three regions; a glycolipid portion (lipid A) covalently linked to an oligosaccharide moiety (core) which is, in turn, linked to an O-polysaccharide chain (O-chain) [12,13]. The LPS is termed smooth (S-LPS) when the O-chain is present, whereas it is termed rough when the O-chain is absent (R-LPS or lipooligosaccharide) [12]. We previously isolated and characterized the lipooligosaccharide (LOS) from the wild sample of *S. vesiculosa* HM13 [14]. Usually, the small amount of EMVs’ biomass does not allow for the isolation of enough amounts of LPS from EMVs to be characterized [15]. However, in contrast to many other bacteria, *S. vesiculosa* HM13 produces a large amount of EMVs which allows detailed characterization of their component molecules. In this study, we characterized the complete structure of the LOS isolated from both the cells and EMVs of *S. vesiculosa* HM13. The structural characterization was performed by mass spectrometry and NMR spectroscopy. The structures of the LOS isolated from the cells and EMVs were compared.

## 2. Results and Discussion

### 2.1. LOS Isolation and Chemical Analysis

*S. vesiculosa* HM13 (rifampin-resistant mutant) was grown in LB medium at 18 °C to the stationary phase. EMVs were collected from the broth culture, as described in the experimental section. The rifampin-resistant mutant was used in this study because it is used as a parental strain for mechanistic studies on the biogenesis of EMVs.

**Figure 1:**
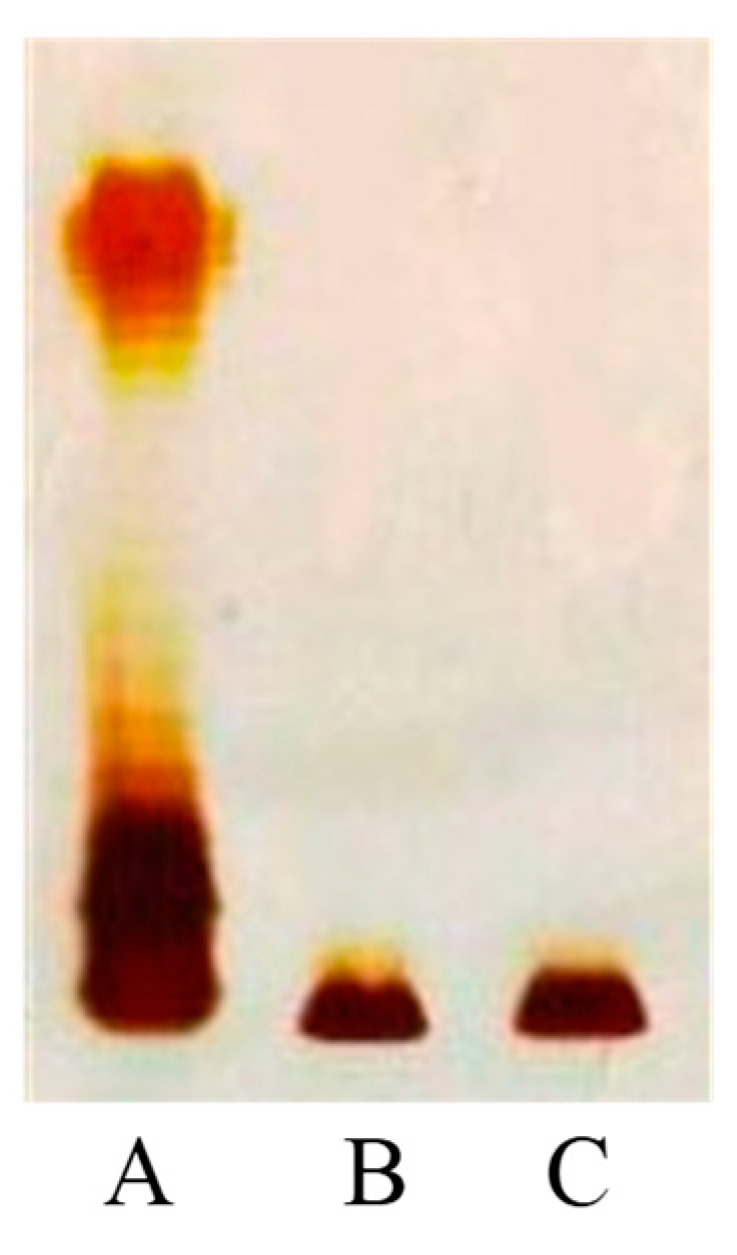
Analysis of the lipooligosaccharide (LOS) from *S. vesiculosa* HM13 rifampin-resistant cells (Lane B) and extracellular membrane vesicles (EMVs) (Lane C) by 14% sodium deoxycholate polyacrylamide gel electrophoresis (DOC-PAGE). The LOSs from the cells and the EMVs were compared with a smooth-LPS from *Escherichia coli* O127: B8 (Lane A).

LOSs of the dried cells and EMVs were extracted using the phenol/chloroform/light petroleum (PCP) method [16]. The LOS yield for the cells and the EMVs after the precipitation was 3% and 19%, respectively. The LOS extracts were analyzed using sodium deoxycholate polyacrylamide gel electrophoresis (DOC-PAGE, Figure 1). After silver nitrate staining, the gel showed for both samples the presence of the bands at low molecular masses, thus confirming the presence of an R-type LPS. The same quickly migrating species was observed for the cells of the bacterium *S. vesiculosa* HM13 grown at 4 °C [14].

**Figure 2:**
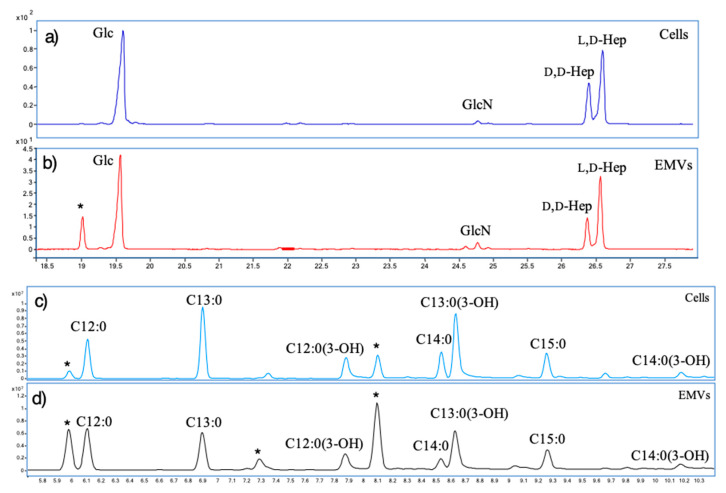
Gas chromatography-mass spectrometry (GC-MS) chromatograms of acetylated methyl glycosides of the LOS samples from *S. vesiculosa* HM13 cells (**a**) and EMVs (**b**). GC-MS chromatograms of the fatty acids methyl esters of the LOS samples from *S. vesiculosa* HM13 cells (**c**) and EMVs (**d**). Asterisks “*” indicate contaminants.

The sugar composition for both LOSs was obtained by gas chromatography-mass spectrometry (GC-MS) of the acetylated methyl glycosides. The GC-MS profiles revealed the same sugar composition as that obtained for the cells of *S. vesiculosa* HM13 grown at 4 °C [14]. In particular, the analysis confirmed the presence of glucose (Glc), 2-amino-2-deoxy-glucose (GlcN), and both d,d- and l-*glycero*-d-*manno*-heptoses (d,d-Hepand l,d-Hep, respectively) (Figure 2a,b, and Table S1, Supporting Information). The fatty acids analysis indicated for both the cells and the EMVs C12:0(3OH) and C13:0(3OH), as the major 3-hydroxylated components (Figure 2c,d, and Table S2, Supporting Information). Additionally, a small amount of C14:0(3OH) was revealed. Non-hydroxylated fatty acid residues were found to be C12:0, C13:0, C14:0, and C15:0.

**Figure 3:**
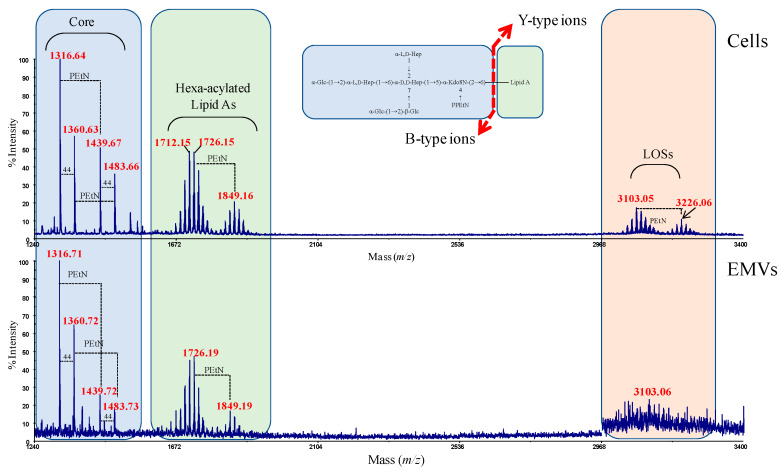
Negative ions MALDI-TOF MS spectra of intact LOSs from the *S. vesiculosa* HM13 cells and EMVs. The insert shows the fragment ions.

The intact LOSs from the cells and EMVs were analyzed in-depth by mass spectrometry analysis. The negative ions MALDI-TOF spectra of the intact LOSs (Figure 3) suggested that both have identical composition, as revealed from the ion at *m/z* 3103.05 (Calculated [M − H]^−^ *m/z* 3103.56). In the same spectra, signals assignable to the lipid A and to the core oligosaccharide (OS) fragments, arising from the in-source cleavage of the linkage between the GlcNII of the lipid A backbone and the 8-amino-3,8-dideoxy-*manno*-oct-2-ulosonic acid (Kdo8N) residue, were detected [14] (Figure 3). The mass spectra revealed that signals attributable to the core fragments were the same as those obtained at 4 °C [14]. Indeed, the signals at *m/z* 1360.63 and 1483.66 can be attributed to the fragments Hex_3_Hep_3_Kdo8NP and Hex_3_Hep_3_Kdo8NPPEtN, respectively (Calculated [M − H]^−^ *m/z* 1360.38 and 1483.39, respectively) (Hex, hexose; Hep, heptose; P, phosphate group; PEtN, phosphoethanolamine group).

### 2.2. Structural Characterization of the Lipid A from Cells and EMVs

To determine the chemical structure of the lipid A moiety from both LOSs (cells and EMVs), a mild acid hydrolysis was performed. The reaction yielded the water-soluble core oligosaccharide in the liquid phase and the lipid A domain as a precipitate.

**Figure 4:**
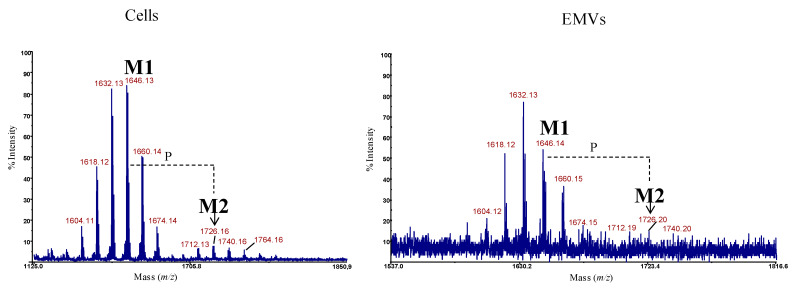
Negative ions MALDI-TOF spectra of the lipid A samples from the *S. vesiculosa* HM13 cells and EMVs. P indicates the loss of the phosphate group.

**Scheme 1:**
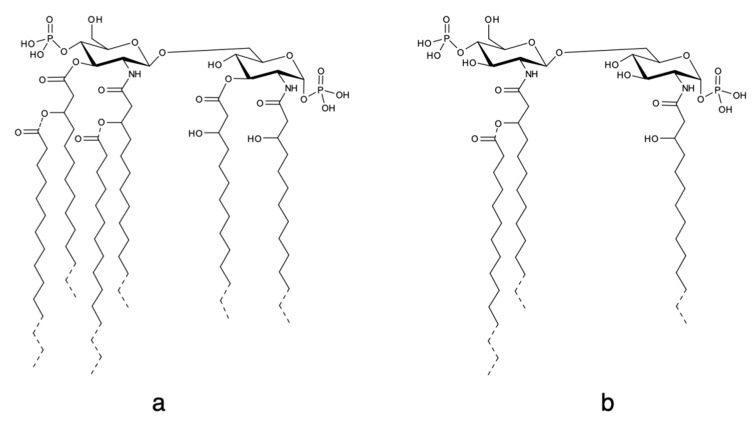
Structure of the lipid A (**a**) and of the product obtained after alkaline hydrolysis with NH_4_OH (**b**).

To establish the distribution of the acyl chains on the glucosamine disaccharide, negative ions MALDI-TOF spectra of the lipid A samples were performed (Figure 4). The spectra indicated, as the major species, two signals clusters referred to as **M1** and **M2**, corresponding to glycoforms with different acylation patterns (Figure 4, Scheme 1a).

Both glycoforms were hexa-acylated, with a difference of 80 Da between the two clusters, each corresponding to a phosphate group. Variations of fatty acid length are responsible for the 14 Da mass differences within each cluster. In particular, to the ion at *m/z* 1726.16, the following composition was attributed GlcN_2_P_2_[C13:0(3-OH)]_3_[C12:0(3-OH)][C13:0]_2_ (Calculated [M − H]^−^ *m/z* 1726.15).

**Figure 5:**
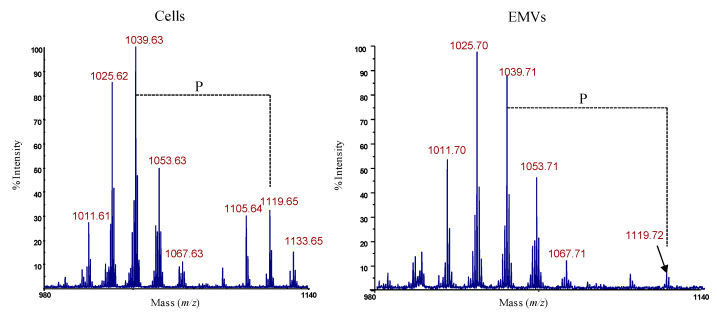
Negative ions MALDI-TOF spectra of NH_4_OH treated lipid As from *S. vesiculosa* HM13 cells and EMVs. P indicates the loss of the phosphate group.

In addition, the mass spectra of the entire LOSs revealed that phosphoethanolamine substituted the lipid A in both structures (Figure 3 and Figure S1, Supporting Information). To detect the position of the secondary fatty acids, an alkaline hydrolysis with NH_4_OH was performed. This procedure obtained a lipid A devoid of acyl and acyloxacyl esters, leaving intact acyl and acyloxacyl amides. The negative ions MALDI-TOF spectra of the samples (Figure 5, Scheme 1b) indicated a pattern of signals that were assigned as follows: the signal at *m/z* = 1119.65 was attributed to a tri-acylated bis-phosphorylated lipid A species with two amide-linked 13:0(3-OH) residues, one of which is substituted at O-3 by a secondary 13:0 acyl chain (Calculated [M − H]^−^*m/z* 1119.62). The differences of 14 Da less can be attributed to a C12:0 as secondary fatty acid as well as to a C12:0(3-OH) instead of a C13:0(3-OH) as primary fatty acid residues. Similarly, the additional 14 Da can be due to a combination of two C13:0(3-OH) residues and one C14:0, as well as a C14:0(3-OH) instead of a C13:0(3-OH) as a primary fatty acid.

Differences in the amount of the double phosphorylated species in comparison with the monophosphorylated ones in both lipid A spectra can be due to a partial hydrolysis of the phosphate group during the acetic acid treatment. Indeed, there are no differences in the relative amount of the intact lipid A species (Figure 3 and Figure S1, Supporting Information). Taking together all these results and comparing them with those already reported for *Shewanella pacifica* [17], we concluded that the lipid A structures were the same between the cells and the EMVs with regard to the acyl substitution pattern. So far, the presence of a phosphoethanolamine group substituting the lipid A of *Shewanella* species has never been described.

### 2.3. De-O-acylation and de-N-acylation of the LOS from the EMVs

**Figure 6:**
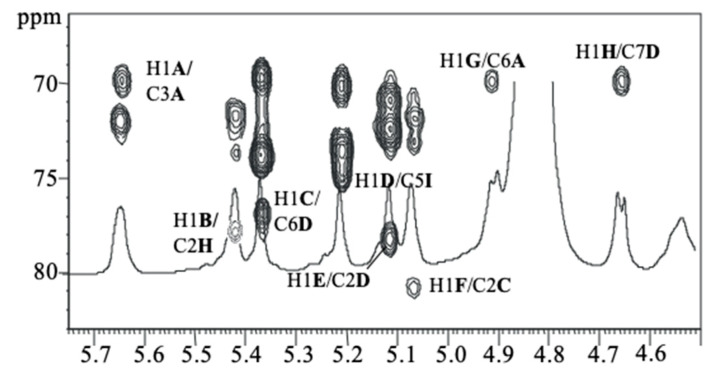
^1^H, ^13^C HMBC anomeric region of the oligosaccharide obtained after de-acylation of the LOS from *S. vesiculosa* HM13 EMVs. The spectrum was recorded in D_2_O at 298 K at 600 MHz. The letters refer to the residues as described in Table 1.

To confirm the structure of the core oligosaccharide region of LOS from the EMVs, a full de-acylation was executed. The LOS from the EMVs was de-*O*-acylated by mild hydrazinolysis and then de-*N*-acylated by strong alkaline hydrolysis [14]. The mixture was desalted on a Sephadex column, yielding the core fraction. To characterize the oligosaccharide attached to the lipid A glucosamine disaccharide, a set of homo- and hetero-nuclear 1D and 2D NMR experiments were performed (^1^H, ^1^H DQF-COSY, ^1^H, ^1^H TOCSY, ^1^H, ^1^H ROESY, ^1^H, ^13^C DEPT-HSQC, ^1^H, ^13^C HMBC and ^1^H, ^31^P HMBC). The ^1^H NMR spectra of the LOS carbohydrate backbone from the EMVs displayed the same eight anomeric signals already found for the oligosaccharide from cells grown at 4 °C (residues A-H, Table 1), despite the slight differences in the chemical shift values. The study of 2D-NMR spectra allowed assigning all the spin systems of the monosaccharides of the core region (Table 1). Among these, the anomeric region of the HMBC experiment was reported in Figure 6. This experiment indicated that the sequence of the oligosaccharide structure from the EMVs was the same as that already found for *S. vesiculosa* HM13 grown at 4 °C [14].

Finally, the ^1^H-^31^P HSQC experiment of OS from *S. vesiculosa* HM13 EMVs indicated correlations for the ^31^P with ^1^H signals as follows: H1 and H2 of GlcNI with ^31^P signal at δ 1.73 ppm; H4 of GlcNII with ^31^P signal at δ 3.47 ppm; H4 of Kdo8N with ^31^P signal at δ 3.77 ppm (Figure S2, Supporting Information). Finally, the correlations of the ^31^P at δ 3.86 ppm with the ^1^H signals at δ 4.00 and 3.24 ppm were attributed to free PEtN molecules [18].

**Scheme 2:**
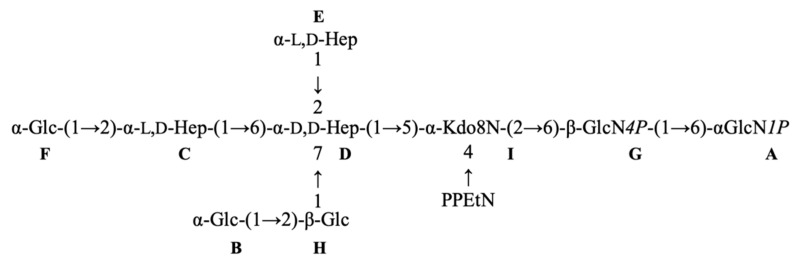
OS structure of the LOS molecule from EMV of S. vesiculosa HM13.

Finally, all the above results made it possible to define the complete structure of the LOS molecule from EMV of *S. vesiculosa* HM13 (Scheme 2).

### 2.4. Possible Involvement of LOS in Interaction of P49 with EMVs and the Cells

We recently found that disruption of the genes coding for putative surface glycolipid biosynthesis proteins, including LOS *phosphoethanolamine transferase*, causes dissociation of P49 from EMVs as well as from the cells [11]. The results suggest that surface glycolipids modified with a phosphoethanolamine group play an essential role in the interaction of P49 with both EMVs and the cells. This speculation is consistent with the results obtained in the present study, showing that the chemical structure of LOS from the EMVs was identical to that from the cells and the LOS molecule was modified with a phosphoethanolamine group. Taken together, it is plausible that the same LOS molecule is required for tethering P49 to both EMVs and the cells. This hypothesis will be examined in future studies by analyzing the LOS structure of the mutants that have defects in the interaction of P49 with EMVs and the cells.

## 3. Materials and Methods

### 3.1. Preparation of the Cells and EMVs of S. vesiculosa HM13

A rifampin-resistant mutant of *S. vesiculosa* HM13 [10] was grown aerobically in LB medium at 18 °C to the stationary phase (OD_600_ = 2.5). The culture was centrifuged at 6800× *g* for 10–20 min at 4 °C to pellet the cells, which were used for LOS analysis after washing with Dulbecco’s phosphate buffered saline. The culture supernatant was centrifuged again at 13,000× *g* for 15 min at 4 °C. The supernatant obtained was filtrated with 0.45-µm membrane filter. The filtrate was concentrated with Minimate^TM^ Tangential Flow Filtration system equipped with a 500K Minimate capsule with Omega membrane (Pall Corporation, Port Washington, NY, USA) at 4 °C. The concentrate was ultracentrifuged at 100,000× *g* for 2 h at 4 °C to collect EMVs for LOS analysis.

### 3.2. LOS Isolation

Dried cells (3.8 g) and EMVs (202 mg) were extracted using the PCP method [16]. The LOS was recovered as a pellet after centrifugation, washed with cold acetone, and freeze-dried.

### 3.3. DOC-PAGE Analysis

Polyacrylamide gel electrophoresis (PAGE) was performed using the system of Laemmli [19] with sodium deoxycholate (DOC) as detergent as already reported [14]. LOSs bands were visualized after silver nitrate staining [20].

### 3.4. Chemical Analysis

The monosaccharides were analyzed as acetylated methyl glycosides by GC-MS [14]. Briefly, an aliquot of LOS samples (0.5 mg) from the cells and EMVs were subjected to a methanolysis reaction with HCl/CH_3_OH (1.25 M, 1 mL) at 80 °C for 16 h. The mixture was extracted three times with hexane. The hexane phases containing the fatty acids methyl esters were directly analyzed by GC-MS. The methanol layers, containing the methyl glycosides were dried and then acetylated with acetic anhydride in pyridine at 100 °C for 30 min.

All the samples were analyzed on an Agilent Technologies gas chromatograph 6850A equipped with a mass selective detector 5973N and a Zebron ZB-5 capillary column (Phenomenex, Bologna, Italy 30 m × 0.25 mm i.d., flow rate 1 mL/min, He as carrier gas). Acetylated methyl glycosides were analyzed using the following temperature program: 140 °C for 3 min, then 140→240 °C at 3 °C/min. The temperature program for methyl esters of fatty acids was the following: 140 °C for 3 min, then 140→280 °C at 10 °C/min, and finally 280 °C for 20 min.

### 3.5. Deacylation of the LOS

The LOS sample from the EMVs (32.9 mg) was dried over phosphorus anhydride in a vacuum chamber and then treated with hydrazine (1.75 mL) at 37 °C for 2 h. Cold acetone was added to precipitate the de-*O*-acylated LOS. The pellet was recovered after centrifugation at 4 °C and 7000 rpm for 30 min, washed three times with acetone, dissolved in water, and lyophilized (23 mg) [21]. The *O*-deacylated LOS (23 mg) was submitted to a reaction with 4 M KOH (1.0 mL) for 16 h at 120 °C. The reaction mixture was neutralized with 2 M HCl (until pH 6) and extracted with CHCl_3_ three times. The aqueous phase was desalted on a Sephadex G-10 column (GE Healthcare, Pittsburgh, PA, USA, 2.5 × 43 cm, 31 mL h^−1^, fraction volume 2.5 mL, eluent 10 mM NH_4_HCO_3_). The eluted fraction was then lyophilized (6.9 mg).

### 3.6. Mass Spectrometry Analysis

MALDI-TOF mass spectra were acquired on an ABSCIEX TOF/TOF™5800 (AB SCIEX, Darmstadt, Germany) mass spectrometer equipped with an Nd:YLF laser with a λ of 345 nm, a < 500-ps pulse length, and a repetition rate of up to 1000 Hz. Approximately 2000 laser shots were accumulated for each spectrum. The calibration of the mass spectra was obtained with a hyaluronan oligosaccharides mixture. A solution of 2,5-dihydroxybenzoic acid (DHB) in 20% CH_3_CN in water (25 mg mL^−1^) was used as the matrix. The samples were desalted on a Dowex 50WX8 (H^+^ form) and dissolved in 2-propanol/water with a 1:1 ratio. The spectra were calibrated and processed under computer control by using Data Explorer software v.0.2.0 (Microsoft, Albuquerque, NM, USA). Mass accuracy was found to be better than 170 ppm.

### 3.7. NMR Spectroscopy

1D and 2D ^1^H and ^13^C NMR spectra were recorded at 298 K with a Bruker Avance 600 MHz equipped with a cryoprobe. The chemical shifts were measured in D_2_O using sodium 3-trimethylsilyl-(2,2,3,3-^2^H_4_)-propanoate (TSP, ẟ_H_ 0.00) as an internal standard for the protons. 1,4-dioxane (δ_C_ 67.40 ppm) was used for the carbon atoms. All 2D homo- and hetero-nuclear experiments (COSY, TOCSY, ROESY, DEPT-HSQC, and HMBC) were performed as already reported [22]. The ^1^H,^31^P HMBC experiment was recorded on a Bruker DRX-400 spectrometer as already reported [23].

## 4. Conclusions

In this study, the LOSs isolated from both the cells and EMVs of *S. vesiculosa* HM13 were fully characterized. The characterization revealed the same structure for the cells and EMVs. Finally, no differences were found with respect to the LOS between the cells grown at 4 °C and 18 °C. Cold-adapted bacteria have been examined for the EMVs’ production and their LPSs’ content [15]. However, to our knowledge, this is the first study about the isolation and the complete characterization of a lipopolysaccharide from EMVs. It has been reported that in *Porphyromonas gingivalis* the lipid A is less acylated in EMVs compared with cells [24]. In *Pseudomonas aeruginosa*, where two different LPSs have been identified, one of them is enriched in the EMVs relative to the cells [25]. Finally, in *Bacteroides fragilis*, the comparison of cellular lipid A and EMVs revealed that they were identical [26].

*S. vesiculosa* HM13 is capable of embedding a large quantity of P49 cargo protein into its EMVs, although it was also found in its cellular fraction [10]. To go deeper into the mechanism of EMV biogenesis and cargo transport, characterization of the LOS isolated from the EMVs was necessary. The identity of the cellular and EMV LOS structures confirmed the budding and pinching off the biogenesis mechanism of the EMVs from the outer membrane [10]. Mutants of *S. vesiculosa* HM13 that have defects in biosynthesis of LOS are under investigation to understand if LOS-P49 interaction is necessary for the loading of P49 into the EMVs.

## Figures and Tables

**Table 1:** ^1^H and ^13^C assignments of the fully deacylated oligosaccharide from the EMVs of *S. vesiculosa* HM13. All the values are referred to sodium 3-trimethylsilyl-(2,2,3,3-^2^H_4_)-propanoate (TSP, δ_H_ 0.00, internal standard) and 1,4-dioxane in D_2_O (δ_C_ 67.40, external standard). Spectra were recorded at 298 K at 600 MHz.

Residue	H1 C1	H2 C2	H3 C3	H4 C4	H5 C5	H6a,b C6	H7a,b C7	H8a,b C8
**A** α-GlcN*p1P*	5.65 92.0 *1.73*	3.35 55.9	3.91 71.5	3.46 71.2	4.21 73.3	3.93–4.36 70.3		
**B** α-Glc*p*	5.43 98.9	3.60 72.7	3.77 73.8	3.50 70.6	4.05 73.1	3.77 72.3		
**C** α-l,d-Hep*p*	5.37 98.6	3.90 82.3	4.11 70.5	3.82 68.5	3.91 75.2	4.07 70.5	3.80–3.75 64.3	
**D** α-d,d-Hep*p*	5.23 101.6	4.09 79.6	4.17 71.7	3.84 68.4	4.07 74.6	4.35 78.1	4.03 71.0	
**E** α-l,d-Hep*p*	5.12 103.5	4.08 71.3	3.85 72.0	3.87 67.7	3.69 73.9	4.06 70.5	3.82–3.69 64.6	
**F** α-Glc*p*	5.08 102.1	3.62 73.1	3.74 74.2	3.50 70.8	4.04 73.2	3.82 62.1		
**G** β-GlcN*p4P*	4.89 100.8	3.07 57.0	3.80 73.6	3.85 74.2 *3.47*	3.76 75.5	3.53 63.5		
**H** β-Glc*p*	4.66 105.1	3.50 77.3	3.59 75.9	3.44 71.1	3.50 78.4	3.76 62.3		
**I** α-Kdo8N*p4P*	n.d.	- 101.2	2.03–2.28 6.4 *3.77*	4.54 70.6	4.36 76.3	3.93 75.0	4.05 67.1	3.21–3.54 44.4

Signals in italic refer to the ^31^P chemical shifts, measured in D_2_O at 298 K at 160 MHz.

## Supplementary Materials

The following are available online at https://www.mdpi.com/1660-3397/18/5/231/s1. **Figure S1**. Selected region (*m*/*z* 1600–2000), indicating the lipid A signals, of the negative ions MALDI-TOF MS spectra of intact LOSs from the *S. vesiculosa* HM13 cells and EMVs. **Figure S2** Expansion of ^1^H-^31^P HSQC spectrum of the OS from *S. vesiculosa* EMVs LOS. The spectrum was recorded in D_2_O at 298 K at 400 MHz.; **Table S1** Molar ratio percentage of monosaccharide residues of the LOS from *S. vesiculosa* HM13; **Table S2** Molar ratio percentage of fatty acids of the LOS from *S. vesiculosa* HM13.
