# Supplementary material for: Detailed Structural Characterization of the Lipooligosaccharide from the Extracellular Membrane Vesicles of Shewanella vesiculosa HM13
Source: Mar Drugs. 2020 Apr 27;18(5):231. doi: 10.3390/md18050231 (PMC7281004; doi:10.3390/md18050231)
Supplement: Supplementary file 1 [file marinedrugs-18-00231-s001.pdf]

Supporting Information

Detailed structural characterization of the lipooligosaccharide from the extracellular membrane vesicles of *Shewanella vesiculosa* HM13

Rossella Di Guida [1], Angela Casillo,[1,2*] Fumiaki Yokoyama[3], Jun Kawamoto[3], Tatsuo Kurihara[3] and Maria Michela Corsaro[1,2*]

[1] Department of Chemical Sciences, University of Naples "Federico II", Complesso Universitario Monte S. Angelo, Via Cintia 4, 80126 Naples, Italy; angela.casillo@unina.it; ross.diguida@gmail.com; corsaro@unina.it

[2] Task Force Blue Italian Growth BigFedII, University of Naples "Federico II"

[3] Institute for Chemical Research, Kyoto University, Uji, Kyoto 611-0011, Japan; yokoyama@mbc.kuicr.kyoto-u.ac.jp; jun_k@mbc.kuicr.kyoto-u.ac.jp; kurihara@scl.kyoto-u.ac.jp;

* Correspondence: angela.casillo@unina.it; corsaro@unina.it; Tel.: +39-081-674149

Table S1. Molar ratio percentage of monosaccharide residues of the LOS from *S. vesiculosa* HM13

	Glc	GlcN	D,D-Hep	L,D-Hep
Tr. (min)	19.57	24.75	26.38	26.60
Cells	49.9%	1.1%	16%	33%
EMVs	55.7%	1.9%	12.1%	30.3%

Table S2. Molar ratio percentage of fatty acids of the LOS from *S. vesiculosa* HM13

	C12:0	C13:0	C12:0(3-OH)	C14:0	C13:0(3-OH)	C15:0	C14:0(3-OH)
Tr. (min)	6.10	6.89	7.87	8.53	8.63	9.26	10.17
Cells	22.8%	24%	10.2%	8%	23%	9%	3%
EMVs	23.6%	21%	10.8%	6%	23.6%	11.3%	3.7%

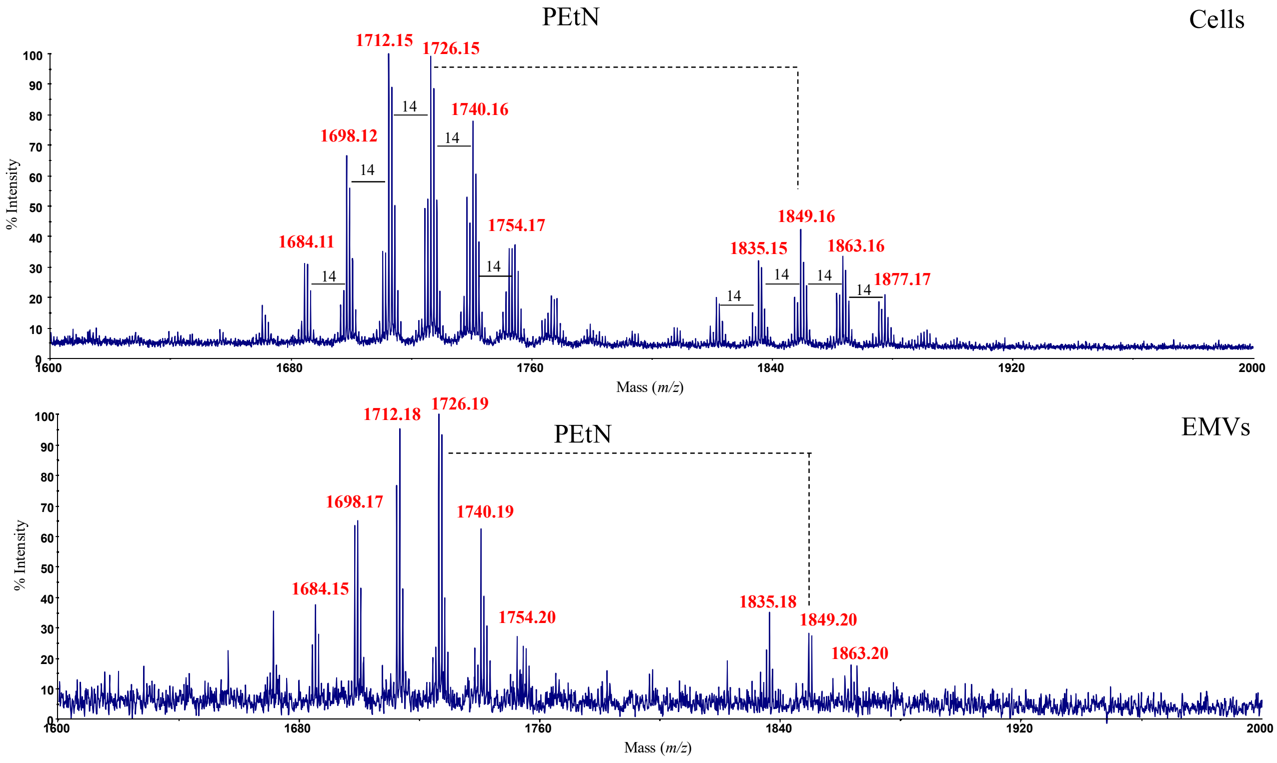

Figure S1. Selected region (*m/z* 1,600-2,000), indicating the lipid A signals, of the negative ions MALDI-TOF MS spectra of intact LOSs from the *S. vesiculosa* HM13 cells and EMVs.

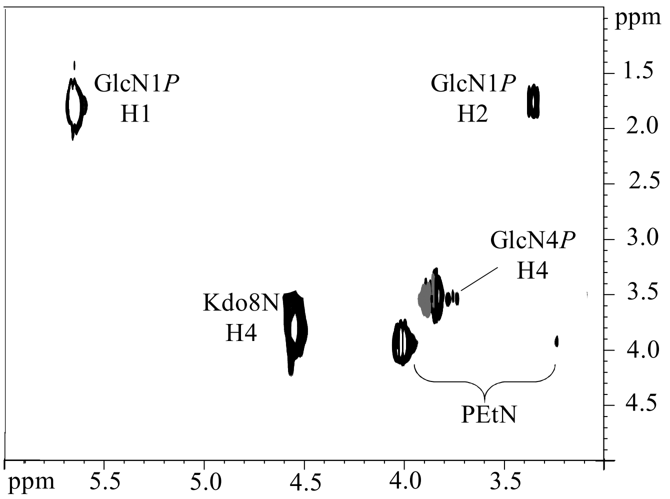

Figure S2. Expansion of ^{1}H-^{31}P HSQC spectrum of the OS from *S. vesiculosa* EMVsLOS. The spectrum was recorded in D_2O at 298 K at 400 MHz.
